# TRPM4 and TRPV2 are two novel prognostic biomarkers and promising targeted therapy in UVM

**DOI:** 10.3389/fmolb.2022.985434

**Published:** 2022-08-23

**Authors:** Jiong Wang, Sen Qiao, Shenzhi Liang, Cheng Qian, Yi Dong, Minghang Pei, Hongmei Wang, Guangming Wan

**Affiliations:** ^1^ Department of Ophthalmology, The First Affiliated Hospital of Zhengzhou University, Zhengzhou, China; ^2^ Assisted Reproduction Center, Northwest Women’s and Children’s Hospital, Xi’an, China; ^3^ Department of Pharmacology, School of Medicine, Southeast University, Nanjing, China

**Keywords:** uveal melanoma, transient receptor potential, transient receptor potential melastatin 4, transient receptor potential vanilloid 2, bioinformatics data analysis

## Abstract

Uveal melanoma (UVM) is the most common primary intraocular malignancy tumor in adults. Almost 50% of UVM patients develop metastatic disease, and is usually fatal within 1 year. However, the mechanism of etiology remains unclear. The lack of prognostic, diagnostic and therapeutic biomarkers is a main limitation for clinical diagnosis and treatment. The transient receptor potential (TRP) channels play important roles in the occurrence and development of tumors, which may have the potential as a therapeutic target for UVM. This current study aimed to identify the potential effect and function of the TRPs that could provide survival prediction and new insight into therapy for UVM. Based on the transcriptome data and potential key genes of UVM were screened using the Cancer Genome Atlas (TCGA) databases, Gene expression analysis showed the expression of TRPM4, TRPV2 and other TRPs was high levels in UVM. Using survival analysis, we screened out that the high expression of TRPM4 and TRPV2 was negatively correlated with the prognosis of UVM patients. Cox regression analysis and functional enrichment analysis further indicated that TRPM4 and TRPV2 were the most convincing therapeutic targets of UVM, and the majority of genes involved in ferroptosis pathways in UVM showed positively correlated with the expression levels of TRPM4 and TRPV2. In conclusion, TRPM4 and TRPV2 were considered as two novel prognostic biomarkers and a promising targeted therapy in UVM.

## Introduction

Uveal melanoma (UVM) is not only the most common primary malignant intraocular tumor in the adult population, but also representing 3.1% of all recorded cases of melanoma (Singh et al., 2011). UVM mainly arises from choroidal melanocytes (90.33%) and less frequently from melanocytes in the ciliary body (6.12%) or iris (3.55%) (Shields et al., 2009). Most patients present between the ages of 50 and 70 years; occurrence before adults is rare (Al-Jamal et al., 2016), risk factors of UVM include fair skin, light eye color, congenital ocular melanocytosis, ocular melanocytoma and the BAP1-tumour predisposition syndrome.

UVM has been divided into two prognostic types according to gene expression profiling of tumor mRNA instead of chromosome status. Class 1 tumors of thoses have a low risk of metastasis, representing almost 50% of the cases, whereas Class 2 tumors of thoses have a high risk up to 72% of metastasis (Onken et al., 2012). Although the treatment options for UVM such as radiation therapy, resection, and enucleation could potentially be curative, up to 50% of patients still develop metastasis regardless treatment with average survival (Bustamante et al., 2021; Toro et al., 2021). Study have showed the median overall survival (OS) of patients with metastatic UVM was 10–13 months for actively treated patients who received systemic therapy, but the surgical resection may be superior but was useful only for a minority of patients (Khoja et al., 2019; Rantala et al., 2019). Furthermore, a study, following over a period of 15 years, described the age of onset for Chinese UVM patients was earlier and meanwhile the prognosis was worse than Western patients (Chen et al., 2022).

Since the rapid development of transcriptome and genome sequencing techniques, it is possible to assess the molecular features of UVM in details such as gene expression profiling and specific gene mutations. Moreover, several studies (Bande Rodriguez et al., 2020; Lamas et al., 2021; Beasley et al., 2022) have attempted to identify the genes which are associated with UVM prognosis. A test named DecisionDx-UM, based on 15 differentially expressed mRNA to predict the risk of UVM metastasis, is used clinically in North America (Plasseraud et al., 2016). Despite the efforts have been made, reliable biomarkers or potential therapeutic targets remain largely unidentified.

Transient receptor potential (TRP) channels are important mediators of sensory perception with significant effects on cellular functions and signalling pathways. Based on sequence homology, the mammalian TRP channel superfamily is classified the majority of TRPs are more selective for calcium, which is considered the most important second messenger involved in physiology and pathological events, such as cancer progression (Morelli and Amantini, 2022; Zhang et al., 2022). Transient receptor potential melastatin 4 (TRPM4) channel is a Ca^2+^-activated nonselective cation channel, belonging to the TRPM family of TRP channels, highly selective for monovalent cations such as Na^+^ and K^+^, but impermeable to anions and divalent cations including Ca^2+^ (Launay et al., 2002; Guo et al., 2017). TRPM4 has been suggested as a protective diagnostic marker for endometrial cancer (Liu et al., 2019). Further studies have shown that the expression of TRPM4 is upregulated in several cancers including breast (Rivas et al., 2020), prostate (Berg et al., 2016) and colorectal cancer (Kappel et al., 2019), and that it contributes to cancer hallmark functions such as increased adhesion, migration, proliferation (Sagredo et al., 2019). However, the mechanism of TRPM4 upregulation in cancer remains elusive.

Transient receptor potential vanilloid 2 (TRPV2) is a non-selective Ca^2+^ permeable cation channel, and is considered to be one of the vanilloid subfamily of TRP channels (Pumroy et al., 2020). TRPV2 is expressed in most organs in the human body and plays a role in phagocytosis in macrophages (Leveque et al., 2018), placental development (De Clercq et al., 2021), T-cell activation (Santoni et al., 2013), and insulin secretion in pancreatic β-cells (Sawatani et al., 2019). TRPV2 has been shown to play an important role in the progression and metastasis of different forms of cancer (Santoni et al., 2020a). Studies have shown that TRPV2 is a promising target for the treatment of prostate cancer and metastasis (Monet et al., 2010; Warrington et al., 2017). TRPV2 activation with 2-APB triggers necrosis and apoptosis in the human metastatic melanoma cell line A2058 (Zheng et al., 2019). Therefore, TRPV2 is a promising target for drug discovery and development of novel therapeutics in cancer. However, the endogenous modulation and pathophysiological function of TRPV2 in cancer remains largely unexplored.

The current study aimed to identify the potential of TRPs as reliable biomarkers for UVM that can predict patient prognosis and responses to therapy. Transcriptome data and potential key genes of UVM were screened using The Cancer Genome Atlas (TCGA) databases. TRP and survival-related genes were evaluated using bioinformatics tools and algorithms. Further analysis of tumor immunologic characteristics and the correlation of ferroptosis with UVM revealed that TRPM4 and TRPV2 are considered as two novel prognostic biomarkers and promising targeted therapy in UVM.

## Materials and methods

### TCGA

TCGA is a publicly available database, covering 80 UVM patient samples, including clinical pathology and bioinformatics data (Hutter and Zenklusen, 2018). We used TCGA database to analyze the TRP expression level in UVM.

### GEPIA

GEPIA (http://gepia.cancer-pku.cn/index.html) is a developed interactive web server for analyzing the RNA sequencing expression data (Tang et al., 2017). In this study, we performed the pathological stage analysis and multiple gene comparison analysis of TRP channel using the “UVM” dataset.

### Kaplan–Meier Plotter

Kaplan–Meier Plotter (https://kmplot.com/analysis/) is a useful prognostic biomarker assessment tool that explores the effect of TRPs on survival in UVM using the databases from TCGA (Nagy et al., 2018). To analyze the prognostic value of TRP channel in UVM regarding overall survival (OS), Disease Specific Survival (DSS), and Progression Free Survival (PFS), the patient samples were split into two groups by the median expression, with the restricted analysis to subtype histology. The hazard ratio with 95% confidence intervals and log-rank *p* value were calculated. We used it to analyze the survival curve of the prognosis of TRP family to verify if the TRP family was related to the prognosis of UVM. Multivariate Cox analysis and Cox proportional hazards regression model were performed on 80 UVM patients. A RS model was constructed using these six genes, and the formula was shown as follows: lambda.min = 0.0662, Riskscore = (0.2368)*TRPV2+(0.5885)*TRPM4+(0.0406)*TRPM8.

### cBioPortal

cBioPortal (www.cbioportal.org) is a comprehensive web resource that could visualize and analyze multidimensional cancer genomics data (Cerami et al., 2012; Gao et al., 2013). Based on the TCGA database, genetic alterations of TRP channel were obtained from cBioPortal, including 80 UVM samples (TCGA, PanCancer Atlas). We used this website to analyze the genetic mutation of TRP family.

### Bioinformatics

Bioinformatics (https://bioinformatics.com.cn) is a data analysis and bioinformatics online analysis website capable of a scientific drawing. In this study, we used this website to perform protein correlation analysis of TRP family.

### UALCAN

UALCAN (http://ualcan.path.uab.edu/analysis.html) is an easy-to-use interactive portal for in-depth analysis of TCGA gene expression data (Chandrashekar et al., 2017). In this study, we used this database to analyze genes correlation related to TRPV2 and TRPM4.

## Results

### The expression pattern of TRPs in UVM

**FIGURE 1:**
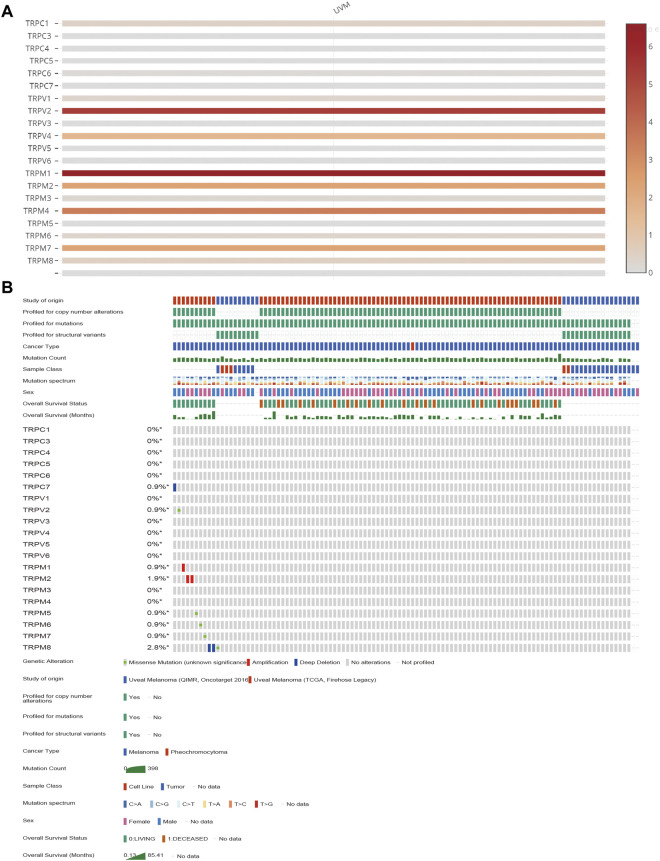
The transcription levels and the genetic alteration analyses of TRPs in UVM patients. **(A)** The heatmap showing mRNA expression levels of TRPs in UVM of TCGA database with the standard bar (right) indicating the relative expression levels. **(B)** The mutations of TRPs in UVM. The mutation levels of TRPs in UVM tissues of TCGA were showen. The mutation probability and mutation types (missense mutation, splice mutation, truncating mutation, structural variant, amplification and deep deletion) of genes were shown.

To determine the expression levels of TRPs in UVM, we extracted all the TRP channel expression data from 80 UVM patient tumour RNA-seq data from TCGA. Since the specialty of UVM, it is infeasible to obtain the normal tissue nearby the tumour. Therefore, we decided to investigate the expression patterns of TRPs only in the tumour tissues. We found the expression levels of TRPC1, TRPC6, TRPV1, TRPV2, TRPV4, TRPM1, TRPM2, TRPM4, TRPM6, TRPM7, and TRPM8 were expressed in UVM tissue. Among these TRPs, TRPV2, TRPM1 and TRPM4 were expressed at relatively high levels in UVM (Figure 1A). This finding was consistent with other previous reports that TRPM8 was considered as a potential drug target for suppressing VEGF induced increases in neovascularization and UM tumor growth by regulating TRPV1 activation (Walcher et al., 2018). Meanwhile, we also investigated the genetic variation of TRPs in UVM. We found that among all 80 samples, one of the sample has deep deletion in TRPC7 locus; one of the samples has missense mutation in TRPV2 locus; one of the samples has amplification in TRPM1 locus; two of the samples have amplification in TRPM2 locus; one of the samples has missense mutation in TRPM5 locus; one of the samples has missense mutation in TRPM6 locus; one of the samples has missense mutation in TRPM7 locus; two of the sample have deep deletion and one of the samples has missense mutation in TRPM8 locus (Figure 1B).

### The prognostic significance of TRPs in UVM patients

**FIGURE 2:**
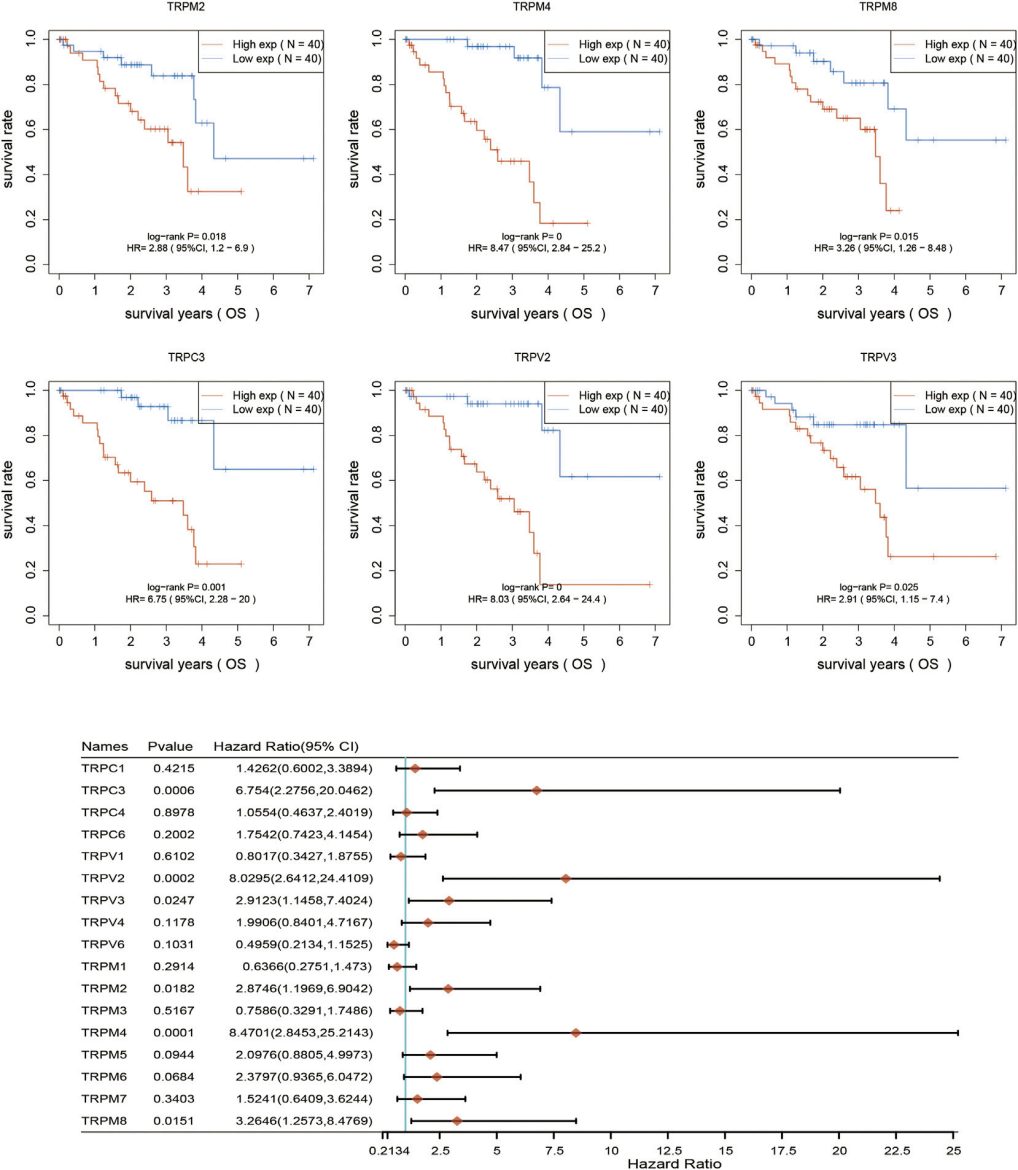
Overall survival curves for the expression of TRPs in UVM patients. The OS curves were performed by using K-M Plotter database. Representations of different samples from low (black lines) and high (red lines) expression groups were shown respectively. + denoted censored observations. The log-rank test was used to compare the OS between groups, with *p* < 0.05 considered statistically significant.

**FIGURE 3:**
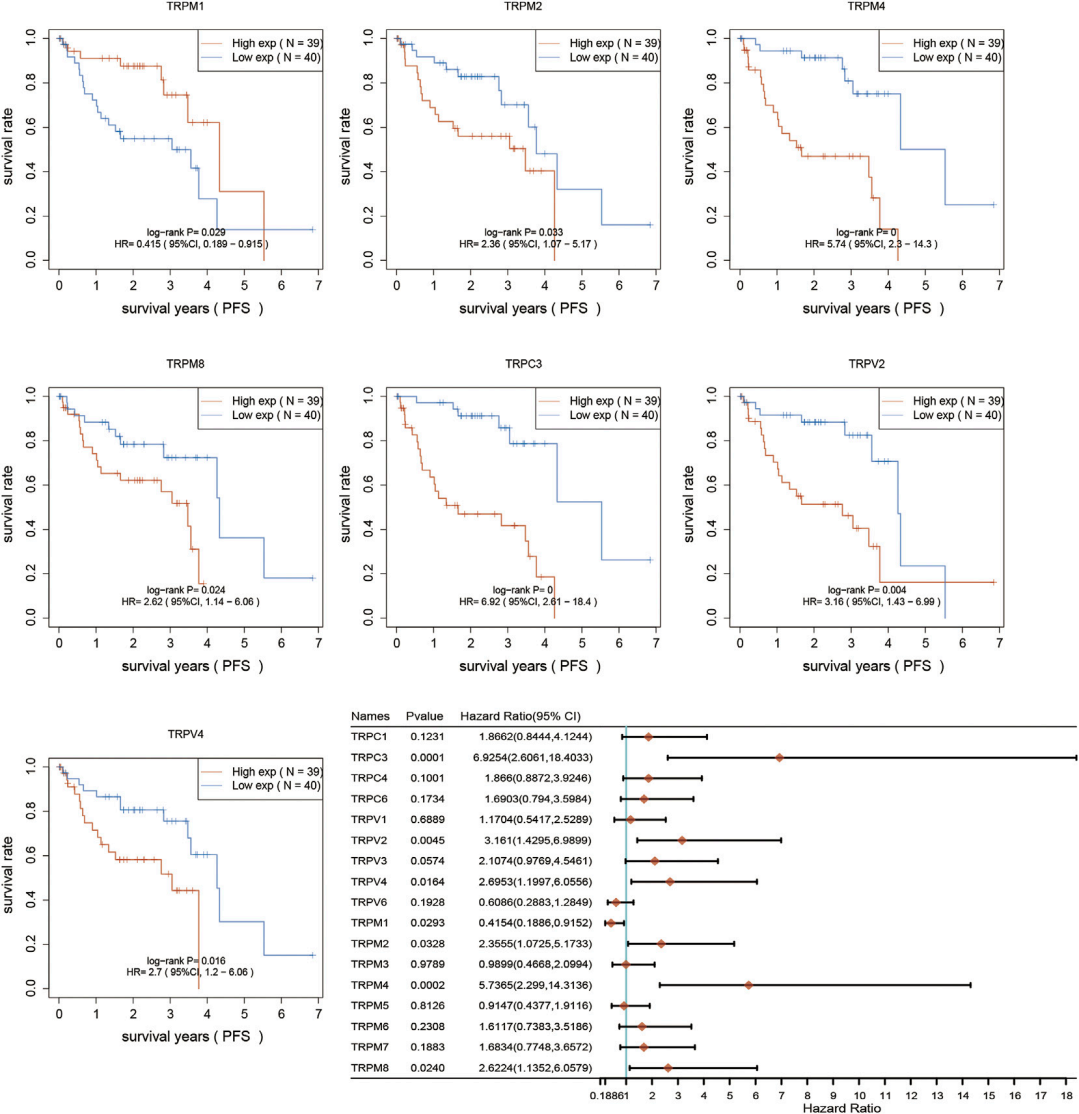
Progression-free survival curves for the expression of TRPs in UVM patients. The Progression-free survival curves were performed by using K-M Plotter database. Representations of different samples from low (black lines) and high (red lines) expression groups were shown respectively. + denoted censored observations. The log-rank test was used to compare the OS between groups, with *p* < 0.05 considered statistically significant.

**FIGURE 4:**
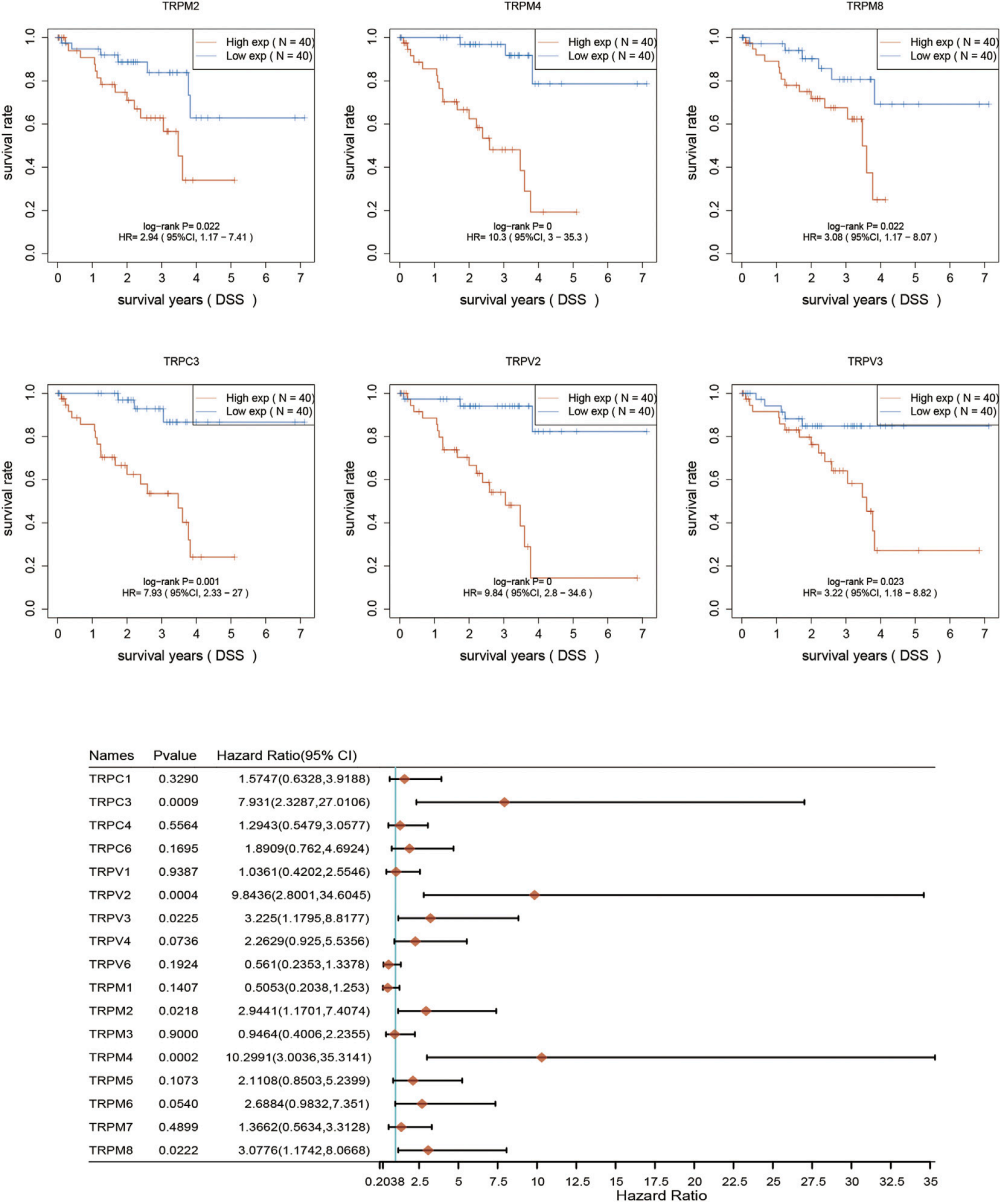
Disease-specific survival curves for the expression of TRPs in UVM patients. The disease-specific survival were performed by using K-M Plotter database. Representations of different samples from low (black lines) and high (red lines) expression groups were shown respectively. + denoted censored observations. The log-rank test was used to compare the OS between groups, with *p* < 0.05 considered statistically significant.

After determination of the expression patterns of TRPs, we wondered that whether the these expressed TRPs had prognostic significance in UVM. To evaluate the prognostic value of TRPs in UVM patients, we performed K-M survival analysis by Kaplan-Meier Plotter. We found the expression levels of TRPM2, TRPM4, TRPM8, TRPC3, TRPV2, and TRPV3 were significantly correlated with OS of UVM. The high expression of these 6 genes corelated with a shorter OS period (Figure 2). Furthermore, we investigated association of TRPs expression patterns with the progression of UVM. We found that the high expression of TRPM2, TRPM4, TRPM8, TRPC3, TRPV2, and TRPV4 was significantly correlated with shorter progression period, meanwhile the high expression of TRPM1 was significantly correlated with longer progression period (Figure 3). Moreover, we investigated association of TRPs expression patterns with disease-specific survival rate of UVM. We found that the high expression of TRPM2, TRPM4, TRPM8, TRPC3, TRPV2, and TRPV3 was significantly correlated with lower DSS rate (Figure 4).

### Univariate and multivariate cox regression analysis in UVM

**FIGURE 5:**
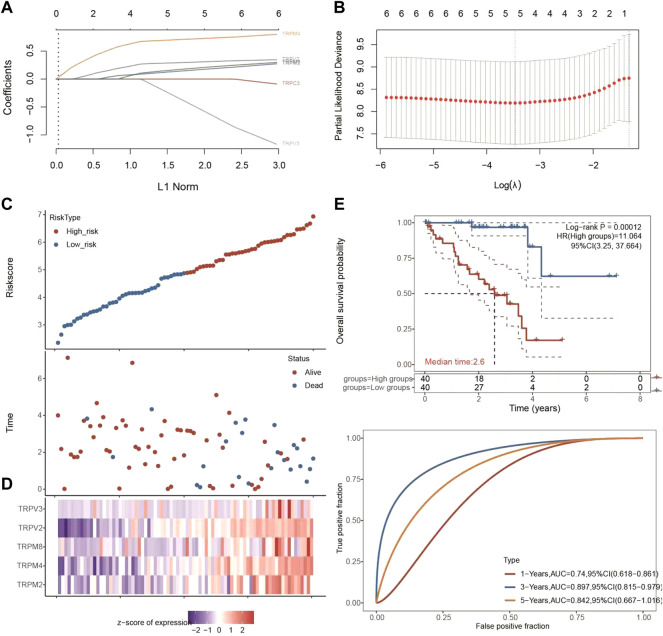
Univariate and multivariate Cox regression analysis of TRPC3, TRPV2/3, and TRPM2/4/8 in UVM patients. **(A,B)** Lasso cox analysis of TRPC3, TRPV2/3, and TRPM2/4/8 in UVM patients. The coefficients of selected features are shown by lambda parameter. The abscissa represents the value of lambda, and the ordinate represents the coefficients of the independent variable. **(C,D)** The riskscore, survival time and survival status of UVM dataset from TCGA. The top scatterplot represents the riskscore from low to high. The scatter plot distribution represents the riskscore of different samples correspond to the survival time and survival status. The buttom heatmap is the gene expression from the signature. **(E)** Kaplan-Meier survival analysis of the risk model from dataset, comparison among different groups was made by log-rank test. The ROC curve and AUC of the gene. The higher values of AUC corresponding to higher predictive power.

Since the expression levels of TRPM4, TRPV2, TRPM2, TRPM8, TRPV3, and TRPC3 were significantly associated with OS of UVM, we then used selected these 6 genes to perform multivariate Cox analysis and construct a Cox proportional hazards regression model from 80 UVM patients. According to this model, we calculated the RS value of each patient and divided the patients into a high-risk group and a low-risk group. We found that the high-risk group had a poor prognosis, indicating these six genes had the potential prognostic ability. Cluster analysis showed that TRPM4 and TRPV2 performed the better potential prognostic ability (Figure 5). The K-M curve showed that OV patients’ OS in the high-risk group was significantly shorter (Figure 5). The three-year ROC curve was the most obvious among 1, 3, and 5-year analyses (Figure 5).

### Functional enrichment analysis of TRP family in UVM patients

**FIGURE 6:**
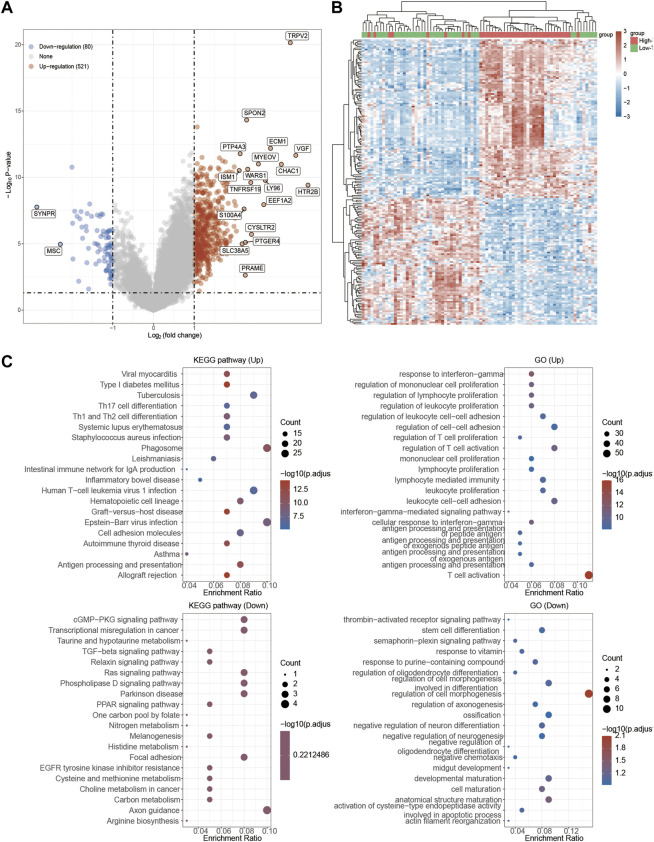
Functional enrichment analysis of TRPV2 in UVM patients. **(A)** Volcano plot: The volcano plot was constructed vis high expressed TRPV2 vs. low expressed TRPV2. Red dots indicate upregulated genes; blue dots indicate downregulated genes; grey dots indicate not significant. **(B)** Heatmap: The heatmap of the differential gene expression, different colors represent the trend of TRPV2 expression in UVM. The top 50 up-regulated genes and top 50 down-regulated genes were showed in this figure. **(C)** Functional enrichment: The enriched KEGG signaling pathways were selected to demonstrate the primary biological actions of major potential mRNA. The abscissa indicates gene ratio and the enriched pathways were presented in the ordinate. Gene ontology (GO) analysis of potential targets of mRNAs. The biological process (BP), cellular component (CC), and molecular function (MF) of potential targets were clustered based on Cluster Profiler package in R software (version: 3.18.0). Colors represent the significance of differential enrichment, the size of the circles represents the number of genes, the larger the circle, the greater the number of genes.

**FIGURE 7:**
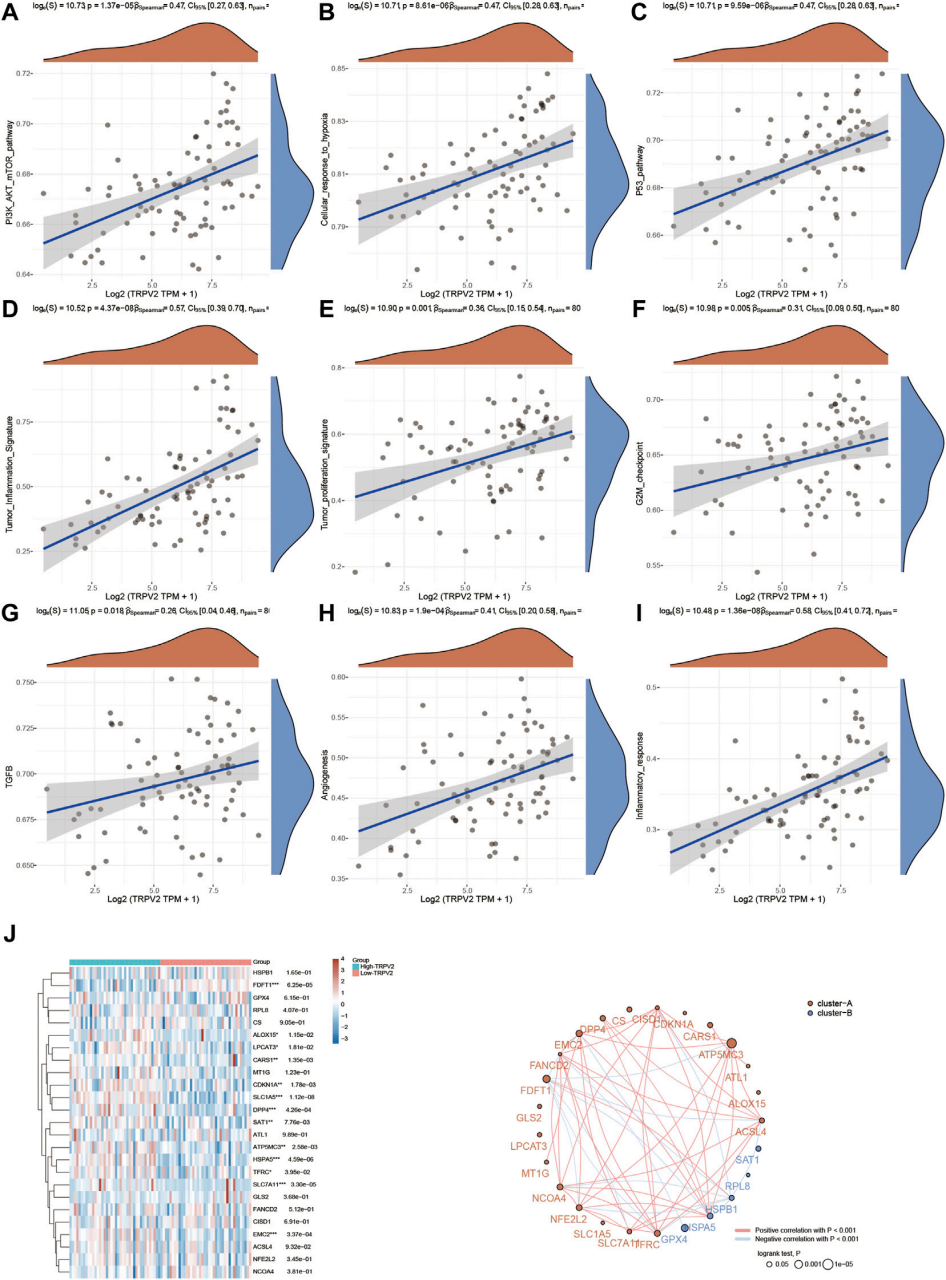
Regulation of functionally relevant pathways by TRPV2 in UVM patients. **(A–I)** The different pathways which were regulated by TRPV2. **(J)** Regulation of the ferroptosis related genes by TRPV2.

Since we found TRPV2 and TRPM4 performed the better potential prognostic ability, we next focused on the associated pathways of these two genes in UVM. For TRPV2, volcano plot showed that most of genes were up-regulated by high expressed TRPV2 compared to low expressed TRPV2 group (FC > 1.5, *p* < 0.05) (Figure 6A). Extracellular matrix protein-1 (ECM1) was reported to promote tumorigenesis in multiple organs (Yin et al., 2021). Overexpression of SPON2 has been shown to promote tumor cell migration by promoting M1/M2-like Macrophage recruitment in colorectal cancer and liver cancer (Zhang et al., 2018; Huang et al., 2021). PTP4A3 was also consider as a prognostic biomarker correlated with immune infiltrates in papillary renal cell carcinoma (Song et al., 2021). In summary, we speculated that high expressed TRPV2 would result to the development of UVM by regulating immune response. The differentially expressed genes were presented in Figure 6B. GO and KEGG analysis were detected through the differentially expressed genes. The differentiation of T helper cells and immune response were significantly up-regulated in Figure 6C. Immune infiltrating cells including lymphocyte, leukocyte, T cell, and monocyte were proliferating in UVM. The cGMP-PKG, TGF-beta, PPAR, and Ras related signaling pathways were down-regulated by high expressed TRPV2 compared to low expressed TRPV2 group. Meanwhile, amino acid related metabolism including histidine, cysteine and methionine, choline, and argine were also down-regulated in UVM. Furthermore, we found that PI3K-AKT-mTOR pathway, cellular response to hypoxia pathway, p53 pathway, tumor inflammation signature, tumor proliferation signature, G2M checkpoint, TGFB pathway, angiogenesis, and inflammatory response pathway were significantly associated with higher expression of TRPV2. Ferroptosis plays an important role in the occurrence and development of tumors, and TRPs act as ion channels in regulating ion in our body. We tried to explore the regulatory mechanism of TRPs in ferroptosis. We found that the expression levels of FDFT1, ALOX15, LPCAT3, CARS1, CDKN1A, SLC1A5, DPP4, SAT1, ATP5MC3, HSPA5, SLC7A11, EMC2 which were involved in ferroptosis pathways, were significantly associated with the expression pattern of TRPV2. Interestingly, the expression levels of the majority of them were positively correlated with the expression levels of TRPV2, which indicated a positive correlation between TRPV2 and ferroptosis in UVM (Figure 7). However, the expression levels of SAT1, RPL8, HSPB1, HSPA5 and GPX4 showed negative correlation with ferroptosis in UVM.

**FIGURE 8:**
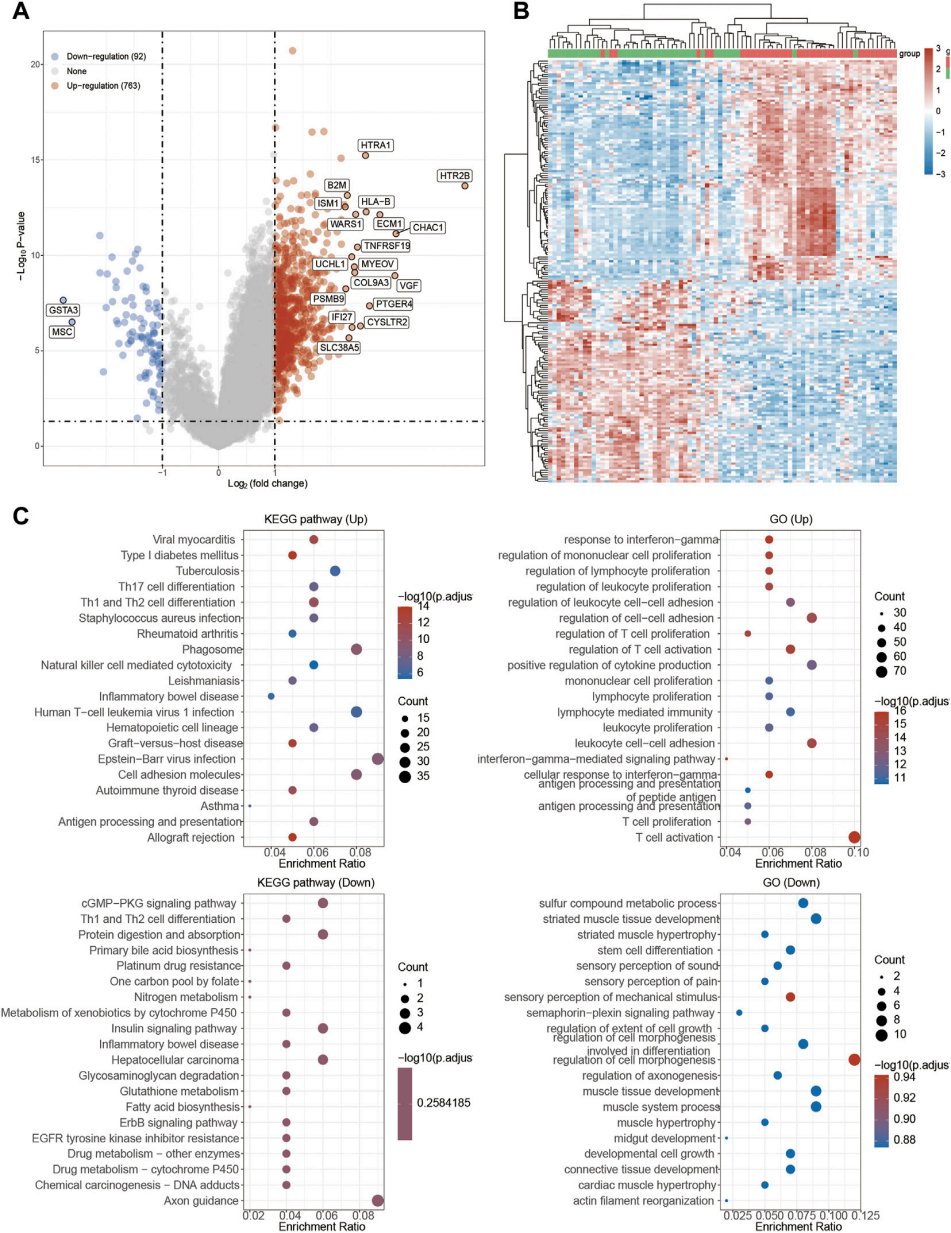
Functional enrichment analysis of TRPM4 in UVM patients. **(A)** Volcano plot: The volcano plot was constructed vis high expressed TRPV2 vs. low expressed TRPM4. Red dots indicate upregulated genes; blue dots indicate downregulated genes; grey dots indicate not significant. **(B)** Heatmap: The heatmap of the differential gene expression, different colors represent the trend of TRPM4 expression in UVM. The top 50 up-regulated genes and top 50 down-regulated genes were showed in this figure. **(C)** Functional enrichment: The enriched GO and KEGG signaling pathways were selected to demonstrate the primary biological actions of major potential mRNA.

**FIGURE 9:**
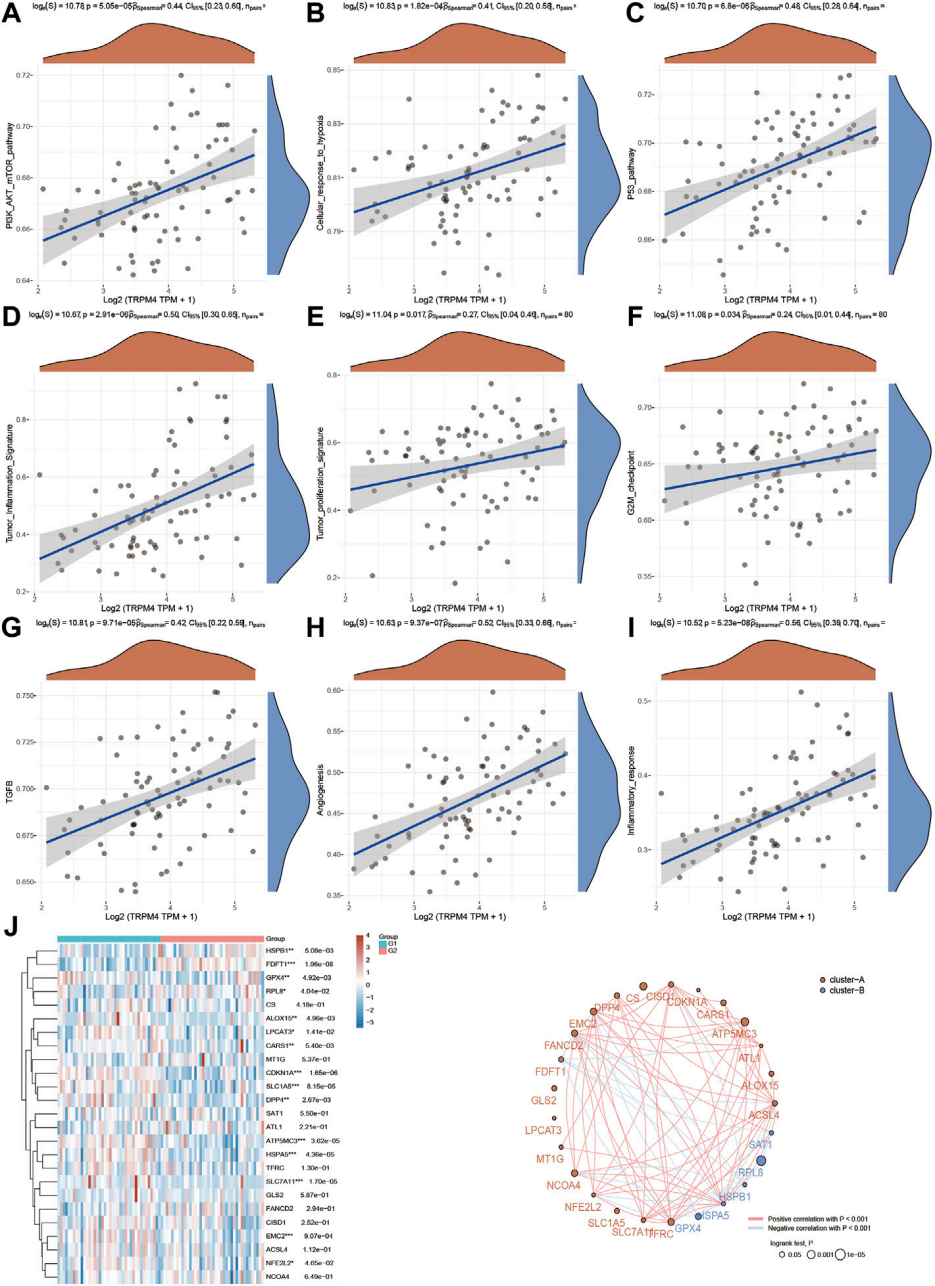
Regulation of functionally relevant pathways by TRPM4 in UVM patients. **(A–I)** The different pathways which were regulated by TRPM4. **(J)** Regulation of the ferroptosis related genes by TRPM4.

Next, functional enrichment analysis of TRPM4 was also measured in Figure 8. There were 763 up-regulated genes and 92 down-regulated genes which was promoted by high expressed TRPM4 (Figure 8A). The differentially expressed genes were also performed by the heatmap (Figure 8B). GO and KEGG analysis showed similar functional enrichment beside that TRPM4 also mainly focus on insulin signaling pathway, lipid metabolism (fatty acid biosysthesis and P450 related pathway), and ErbB signaling pathway. Similarly to pathways associated with TRPV2 in UVM, we found that the expression levels TRPM4 were significantly associated with the pathways mentioned above. We also found the expression levels of the majority of genes involved in ferroptosis were positively correlated with the expression levels of TRPM4 (Figure 9).

### The top associated genes associating with TRPV2 and TRPM4 in UVM

**FIGURE 10:**
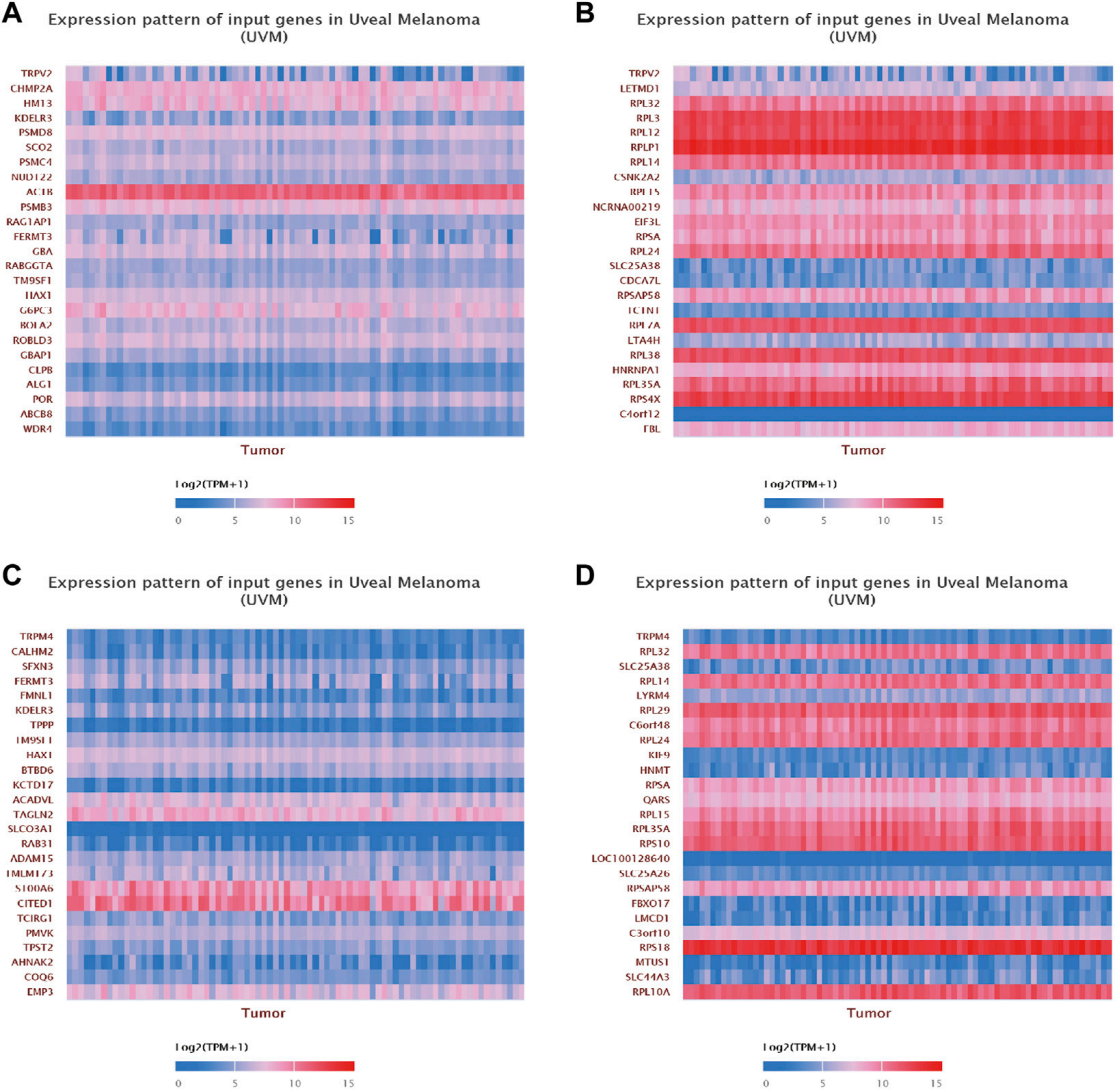
Gene correlation analysis of TRPV2 and TRPM4 in UVM. **(A,B)** The top 25 up-regulated and down-regulated genes related to TRPV2 were shown in the heatmap. **(C,D)** The top 25 up-regulated and down-regulated genes related to TRPM4 were shown in the heatmap.

Finally, we focused on the expression patterns of individual genes which were highly associated with the expression levels of TRPV2 or TRPM4. We found CHMP2A, HM13, KDELR3, PSMD8 and SCO2 were positively associated with the expression of TRPV2, while LETMD1, RPL32, RPL3, RPL12 and RPLP1 were negatively associated with the expression of TRPV2. We found CALHM2, SFXN3, FERMT3, FMNL1 and KDELR3 were positively associated with the expression of TRPM4, while RPL32, SLC25A38, RPL14, LYRM4 and RPL29 were negatively associated with the expression of TRPM4 (Figure 10).

## Discussion

In current study, we found several TRP family members were expressed in UVM tissues with higher expression levels of TRPV2, TRPM1 and TRPM4 than other TRP channel superfamily members. Furthermore, we found that TRPM4 and TRPV2 performed the better potential prognostic ability in UVM. In addition, TRPM4 and TRPV2 were significantly associated several genes and pathways such as ferroptosis pathways in UVM.

UVM arise from melanocytes in the pigmented uveal tissues of the eye, and typically presents with symptoms such as blurred or distorted vision, loss of visual field, or decreased vision; 30.2% of patients are asymptomatic and are therefore only diagnosed during routine examinations (Damato and Damato, 2012). Ophthalmic treatments aimed at conserving the eye and useful vision, preventing metastases if possible, consists of various forms and combinations of radiotherapy, phototherapy and local resection, reserving enucleation in advanced cases (Jager et al., 2020). Currently, effective therapies to prevent the development of metastases are not available, but early treatment of indeterminate lesions may help to prevent the development of lethal UVM (Rantala et al., 2022). However, long-term survival is rare except in patients with isolated liver metastases that are amenable to surgical resection. Once metastasis occured, median progression-free survival (PFS) and OS were 3.3 months and 10.2 months, respectively (Khoja et al., 2019).

UVM is also notable for having two sets of driver mutations, with each tumor typically containing one mutation from each set (Decatur et al., 2016). One group consists of mutually exclusive gain-of-function mutations in Gq signaling pathway members -GNAQ, GNA11, CYSLTR2, or PLCB4. These “Gq mutations” are present in virtually all UVMs, have no prognostic significance and alone are not sufficient to induce malignant transformation, but seemingly required to initiate tumorigenesis (Van Raamsdonk et al., 2009; Van Raamsdonk et al., 2010; Johansson et al., 2016; Moore et al., 2016). The other group consists of near-mutually exclusive mutations in BAP1, SF3B1, and EIF1AX that strongly predicted metastatic risk. Inactivating mutations in the tumor suppressor BAP1 were associated with class 2 and high risk of metastases (Harbour et al., 2010), whereas single nucleotide substitutions in SF3B1 and EIF1AX were predominantly found in class 1 tumors and were associated with intermediate and low risk of metastases, respectively (Harbour et al., 2013; Martin et al., 2013). We also found several TRPs were mutated in UVM tissues, however, due to the low mutation levels, it is tempting to spaculate that these mutations were the results of the tumor rather than the cause of the tumor.

Ca^2+^ ions are key second messengers in excitable and non-excitable cells. However, due to the pleiotropic effects of Ca^2+^ transporters and other Ca^2+^-binding proteins, however, Ca^2+^ signaling has been identified as a potential target of anticancer therapy (Marchi et al., 2020). A major group of membrane-associated Ca^2+^-channels are formed by TRP family proteins that can trigger the activation of specific intracellular cascades through subtle changes in ion influx in response to a variety of extracellular signals (Santoni et al., 2020b). Many TRP channel proteins have shown potential as therapeutical targets for cancer (Morelli and Amantini, 2022). Antagonism of TRPM2 channel leads to antitumor effects in human melanoma cells, including those that are potentially unresponsive to current treatments due to the expression of drug resistance genes (McKamey et al., 2022). TRPM2 represents a potentially novel, efficacious and readily accessible treatment option for patients with melanoma. Transient receptor potential canonical 3 (TRPC3) is widely expressed in human melanoma. Pharmacological inhibition of TRPC3 by a pyrazole compound of Pyr3, decreased melanoma cell proliferation and migration, which indicated that TRPC3 plays an important role in melanoma growth, and may be a novel target for the treatment of melanoma in patients (Oda et al., 2017).

Higher expression levels of TRPM4 and TRPV2 in UVM were significantly associated with pro-cancer signaling pathways, such as P13K-AKT-mTOR signaling pathway, hypoxia, p53 pathway, TGFβ signaling pathway, G2M checkpoint, angiogenesis, proliferation, inflammation and inflammatory response. Inhibition of the PI3K/AKT/mTOR pathway has been reported to be strongly suggested as a therapeutic potential for the prevention and treatment of uveal melanoma (Farhan et al., 2021). *In vitro* study demonstrated that silencing of TRPM4 caused p53 reduction and hyperactivation of EMT, PI3K/AKT/mTOR signaling pathway in endometrial carcinoma, suggesting that TRPM4 can be used clinically to predict endometrial carcinoma prognosis and represents a as potential candidates for new therapeutic targets (Li et al., 2020).

Manipulating Ca^2+^ homeostasis offers a compelling strategy to balance cellular lipids and cell survival in ferroptosis-associated tumors. Ferroptosis, an iron-dependent form of nonapoptotic cell death, has been reported to inhibit or promote tumorigenesis in different models. Meanwhile, we found that the expression patterns of FDFT1, SLCIA5, HSPAS, SLC7A11 and EMC2 were significantly correlated with the expression levels of TRPM4 and TRPV2 in UVM, indicating that TRPM4 and TRPV2 were significantly positively associated with the ferroptosis pathway. Conditional knockout of SLC7A11 induces pancreatic cancer growth, in partly through cysteine depletion-induced ferroptosis (Badgley et al., 2020). Meanwhile, SLC7A11 has been identified as a useful marker to predict the susceptibility of metastatic melanoma cells to ferroptosis (Gagliardi et al., 2019). SLC1A5 (Solute carrier family 1 member 5), a ferroptosis-related gene in the ceRNA network, is a major glutamine transporter that plays a key role in tumor growth, therefore, represents a useful prognostic biomarker in a variety of cancers, and its expression highly correlated with tumor immune-cell infiltration, especially in HCC and LGG (Zhu et al., 2022). By directly targeting the glutamine transporter SLC1A5, miR-137 negatively regulates ferroptosis in melanoma cells and is considered a potential therapeutic approach for melanoma (Luo et al., 2018). In addition, we investigated genes highly associated with TRPV2 and TRPM4 in UVM. Notably, the expression of KDELR3 was highly positive, and the expression of RPL32 was highly negatively correlated with the expression of TRPV2 and TRPM4. (Figure 8). KDELR3, a member of the KDEL receptor family, has been reported as a genuine melanoma metastasis progression gene. KDELR3 is involved in the glycosylation of the metastasis suppressor KAI1 and is degraded by the E3 ubiquitin protein ligase gp78, providing a mechanism by which KDELR3 affects the metastatic phenotype (Marie et al., 2020).

Comparing with other proposed therapotic targets of UVM, such as TRPV2 and TRPM4 have unique advantenges. TRPV2 and TRPM4 are mostly expressed (TRPV2 under stimulation) on the cell membrane, which make these two interesting targets. Membrane expressed proteins can be easier targeted by larger molecules, such as antibody-based drugs. Meanwhile small molecular inhibitors, which can diffuse into cells and thus targeting intracellular signalling. TRPV2 and TRPM4 antagonists were developed, and had been used in *in vitro* studies (Borgstrom et al., 2021; McKamey et al., 2022). With these tools, it is possible to further treat UVM with targeting TRPV2 and TRPM4.

UVM is the most common and fatal intraocular cancer in adults worldwide. Therefore, reliable predictive and prognostic biomarkers must be discovered to improve risk prediction and guide tailored therapy. As with all tumor types, changes in Ca^2+^ channel regulation may contribute to the development and progression of this pathological condition. TRPs are one types of Ca^2+^ permeation pathways that can be dysregulated during tumorigenesis, that have been identified as crucial in tumor development and progression. The identification of functional TRPM4, TRPV2 expression in UVM may provide new drug targets for the treatment of this aggressive tumor disease.

## Data Availability

The original contributions presented in the study are included in the article/Supplementary Material, further inquiries can be directed to the corresponding authors.
